# Supplementary material for: Secretome from Magnetically Stimulated Muscle Exhibits Anticancer Potency: Novel Preconditioning Methodology Highlighting HTRA1 Action
Source: Cells. 2024 Mar 5;13(5):460. doi: 10.3390/cells13050460 (PMC10930715; doi:10.3390/cells13050460)
Supplement: Supplementary file 1 [file cells-13-00460-s001.zip › cells-2875104-supplementary.pdf]

Supplementary Figures

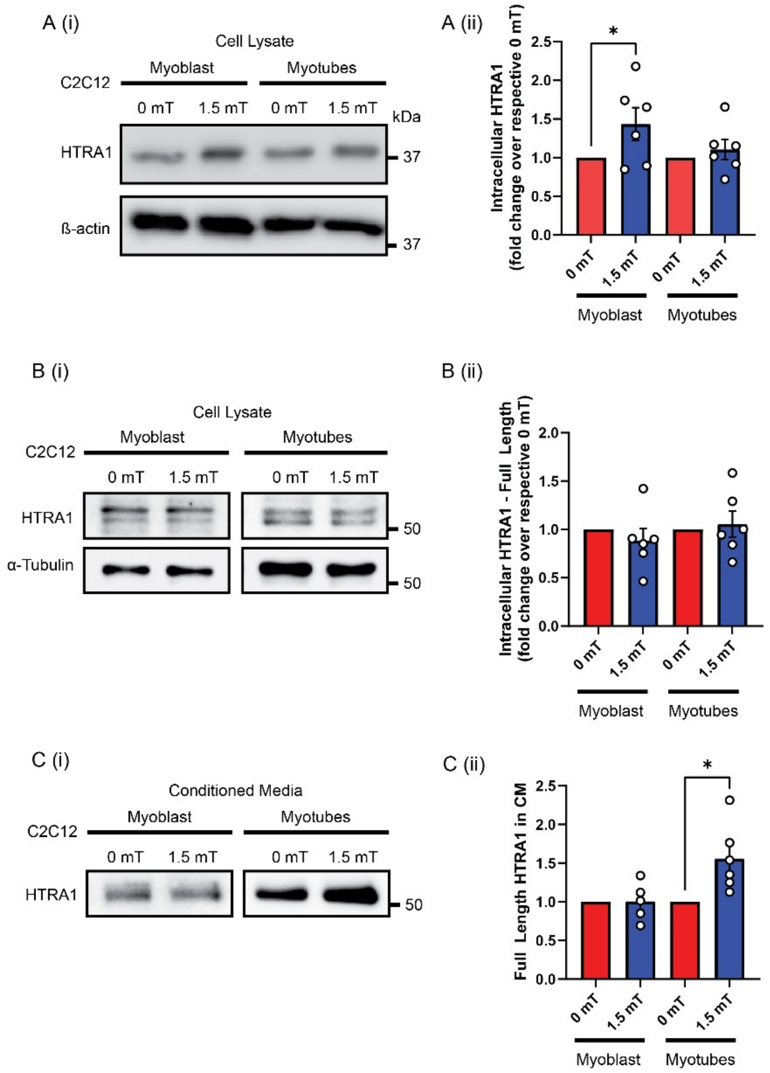

Figure S1. Magnetic induction of full-length and cleaved HTRA1. (A) (i) Immunoblot showing the relative abundance of cleaved HTRA1 (~37 kDA) detected in myoblasts and myotubes from whole cell lysates 24 h post PEMF exposure. (A) (ii) Bar chart of HTRA1 in myoblasts and myotubes expressed as fold change relative to their respective 0 mT controls. (B) (i) Immunoblot showing the relative abundance of full-length HTRA1 (~55 kDA) detected in myoblasts and myotubes from whole cell lysates 24 h post PEMF exposure. (B) (ii) Bar chart of HTRA1 in myoblasts and myotubes expressed as fold change relative to their respective 0 mT controls. C (i) Immunoblot showing full-length HTRA1 levels detected in conditioned media (CM) of myoblast and myotubes 24 h post PEMF exposure. C (ii) shows the relative abundance of HTRA1 in the CM of myoblasts and myotubes. Statistical analysis was performed minimally in five independent experiments, with *p<0.05. The error bars represent the standard error of the mean. Each dot represents independent experiments.

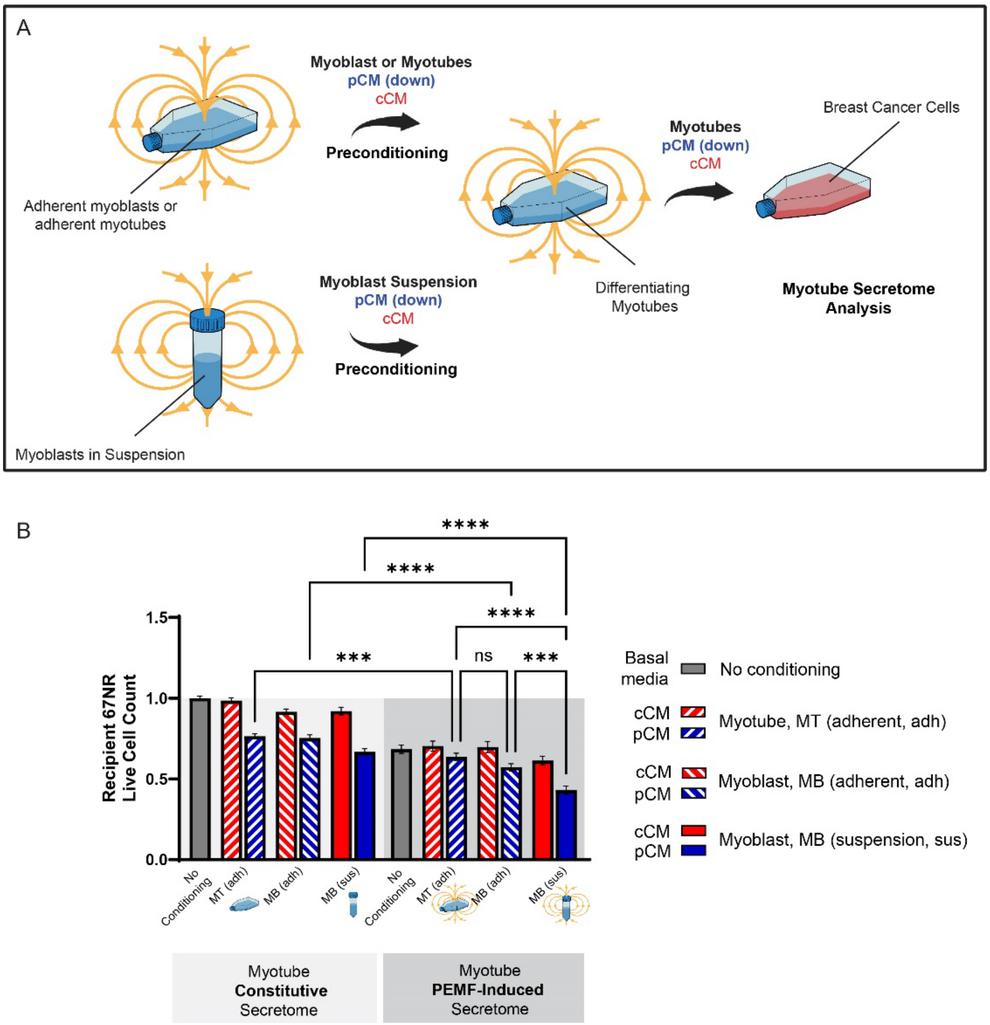

Figure S2. Optimal secretome responses occur with preconditioning with pCM generated from myoblasts in suspension with media conditioning of one hour. (A) Schematic of the preconditioning paradigm. (B) Secretome-modulated 67NR breast cancer cell growth. Secretomes were collected from myotubes in response to the indicated combinations of pCM preconditioning and direct PEMF exposure (dark grey box), or not (light grey box). Cancer cell quantification was conducted 24 h after the provision of the indicated myotube-conditioned media (see Panel A). All presented values were normalized to the response of secretome collected from myotubes differentiated in unexposed myoblast CM in suspension (Figure S2B, first grey bar). Statistical analyses were performed in 3 independent biological repeats of three technical replicates, with ***p<0.001, and ****p<0.0001. The error bars represent the standard error of the mean.

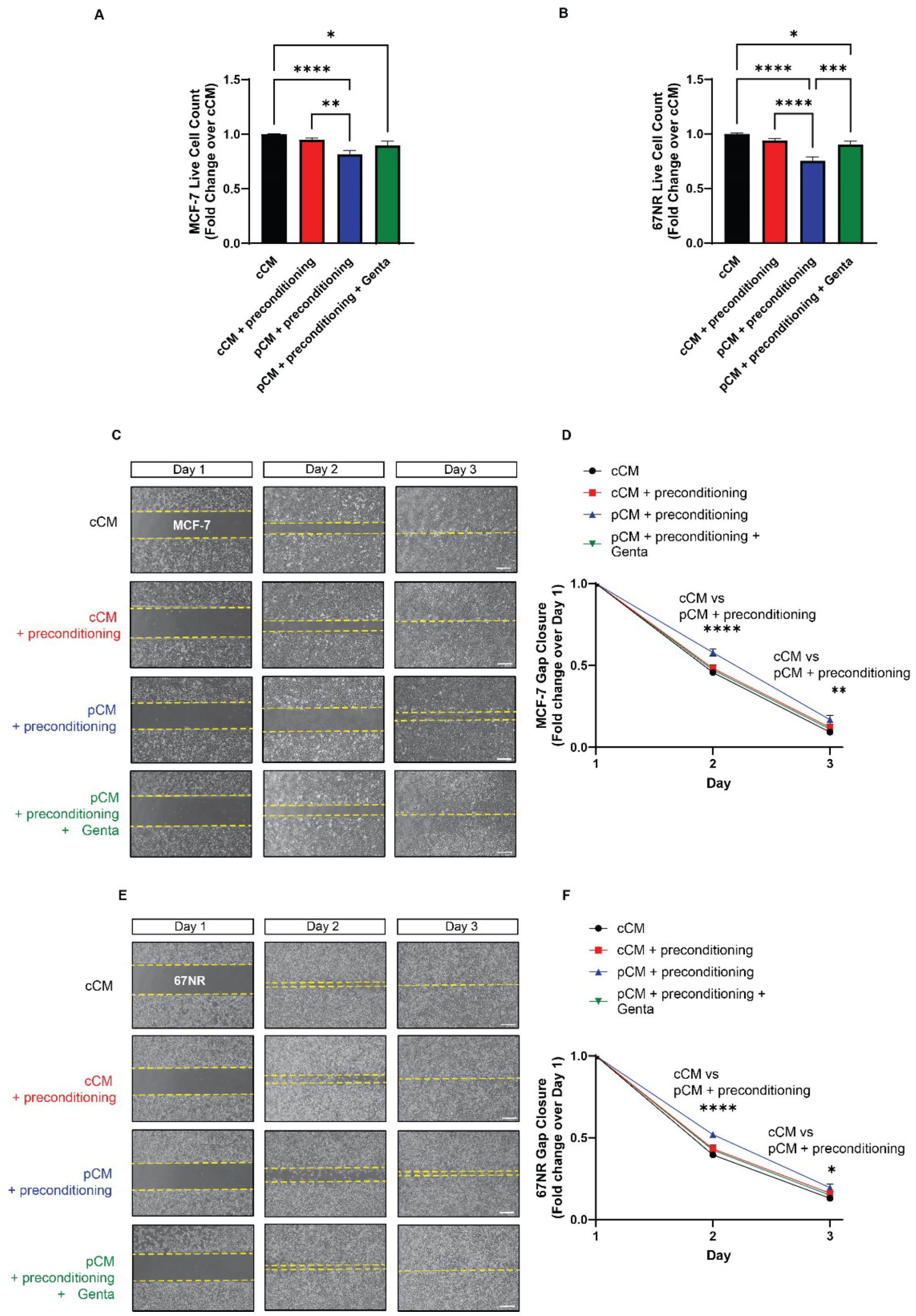

Figure S3. The aminoglycoside antibiotics preclude pCM anticancer efficacy. (A) Quantification of MCF-7 and (B) 67NR breast cancer cells 24 h after the provision of indicated CM. Data is expressed as fold change over cCM (n=3, with 3 technical replicates). (C) Brightfield images showing gap closure over 3 days following the administration of the indicated CM 24 h after the plating of MCF-7 cells. (D) Fold change in MCF-7 cell gap closure in response to the indicated CM over 3 days. (E) Brightfield images showing 67NR cell gap closure over 3 days. (F) Fold change in 67NR cell gap closure in response to the indicated CM over 3 days (n=3). Gentamicin accelerates gap closure in the presence of pCM, which would not be expected if the effect was mediated by an inhibition of protein synthesis.

Statistical analyses were performed with *$p<0.05$, **$p<0.01$, ***$p<0.001$ and ****$p<0.0001$. The error bars represent the standard error of the mean. Scale bar = 300 µm.

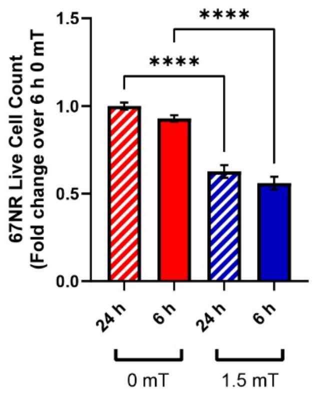

Figure S4. Correlation of anticancer potency in muscle cCM and pCM with conditioning time. The bar chart shows the growth of 67NR breast cancer cells in response to cCM (red) or pCM (blue) at 6 or 24 h post-PEMF conditioning. Statistical analysis was performed on four independent experiments, with ****$p<0.0001$. The error bars represent the standard error of the mean.

Supplementary Methods

Chicken Chorioallantoic Membrane (CAM) Model

Fertilized chicken eggs (Bovan Goldlines Brown) were purchased from Lian Wah Hang Pte Ltd and placed in an egg 38.5°C incubator (Rcom MX50 Automatic incubator) with 70% humidity on Embryonic Day (ED) 0. On ED 3, 3 ml of albumin was removed using an 18G needle in an effort to "lower" the CAM, away from the shell allowing the opening of an access window without injuring the CAM. A 1 cm^2 window on the shell was created and sealed with Tegaderm™ semi-permeable membrane. On ED 6, MCF-7 cells in culture were harvested and pelleted down. Cells were resuspended in Matrigel (Corning) at 1 million cells per 50 µl Matrigel for inoculation. The periderm of the CAM was punctured with a blunt glass rod and 50 µl Matrigel cell suspension was pipetted onto the punctured site. An initial 3D ultrasound was performed on ED 10 to measure the basal tumour size and vascularity. 20 µl of muscle CM was delivered onto a filter paper and placed adjacent to each tumour. The 10X CM was concentrated using Amicon Ultra 15 ml Centrifugal Filters (Merck). Muscle CM was topped up daily for 4 consecutive days. A final 3D ultrasound scan was performed on ED 15 to assess tumour size and vasculature. At the end of the experiment, the CAM tumours were carefully excised from the CAM and weighed.

Ultrasound assessment of tumour on CAM

CAM-engrafted microtumour size and vascularity was assessed by ultrasound. 3D ultrasound was performed on the eggs to measure the tumour size and vascularity using a Fujifilm VisualSonics Vevo 2100 Imaging system, with a 40 MHz centre frequency-MS-550D transducer. Imaging was performed before the initial CM provision and 24 h after the last CM administration. Image analysis was done using the Vevo Lab 1.7.0. A 3D reconstructed image of each tumour was computed from multiple 2D sections obtained. At the time of imaging, the Tegaderm™ transparent film was removed from the egg and a cling wrap was placed gently over the exposed tumour. Care was taken to ensure good contact between the tumour and the cling wrap. The egg is then fixed firmly onto a stage, which scans multiple 2D cross-sections to be reconstructed into a 3D image. Warmed Aquasonic® 100 Ultrasound transducer gel was placed over the cling wrap and the MS-550D transducer was lowered until contact with the gel was established. Ultrasounds in 3D Power Doppler mode are taken at the multiple cross sections at low 2D gain (5 Hz) and Power Doppler gain (10 Hz) using the automated 3D motorized slider. Image analysis was done using the 3D analysis software on Vevo Lab 1.7.0. The circumference of the tumour is carefully traced for the different sections and the tumour volume as well as the percentage vasculature is calculated by the software. Imaging was

performed before the first CM administration and immediately before tumour harvesting, 24 h after the last CM administration for comparison.

Animal study protocol with PEMF treatment and exercise regime

A total of 105 C57BL/6J mice were randomized into 3 groups, with each group comprising 35 mice where the groups were i) Group 1 (Control group – No exercise nor PEMF), ii) Group 2 (PEMF only) and iii) Group 3 (Exercise only). For easier animal handling, the order of mice arrived in 2 separate batches where the earlier batch contained 48 mice and the later batch contained 57 mice. For both batches, the protocol was carried out in the same manner.

Before the start of the study, mice were acclimatized to their cages for a week after randomization. During the same first week of acclimatization, mice in Group 3 were also acclimatized to the treadmill with 2 walking sessions where they were put on a stationary treadmill for 5 min, followed by 10 min of walking at a pace of 6 m per min.

Succinctly, exposure to 1.5 mT amplitude PEMFs for 10 min applied once a week was best suited to promote *in vitro* myogenesis [1]. In this animal study, an analogous PEMF exposure paradigm was employed where specifically, C57BL/6J mice in Group 2 were exposed once per week on Wednesdays for 8 weeks for 10 min at an amplitude of 1.5 mT. The entire mouse was exposed in the PEMF coil system. Individual mice were placed in a clean glass beaker and placed in the middle of the PEMF device. Mice were neither sedated nor anaesthetized for PEMF exposures and were returned to their respective cages after treatment.

The Graded Maximal Exercise Test (GXTm) protocol with modification was adapted for C57BL/6J mice in Group 3 [2]. Instead of an electrical grid shock used to encourage treadmill running, auditory stress was implemented with the aid of paper towels which were placed between the inactivated shock grids such that they would interact with the rotating belt of the treadmill and create noise (Columbus Instruments, Ohio, USA) while the treadmill was in operation, and the noise created was found to be sufficient enough to encourage the mice to run before they reached exhaustion.

The exercise regime was done twice a week every Monday and Friday for a total of 8 weeks (total of 16 sessions) under application number R18-0313 and were as follows with regards to speed, duration, and inclination:

a. 0 m/min; 3 min; 0 deg

b. 6 m/min; 2 min; 0 deg

c. 9 m/min; 2 min; 5 deg

d. 12 m/min; 2 min; 10 deg

e. 15 m/min; 2 min; 10 deg

f. 18 m/min; 1 min; 15 deg

g. 21 m/min; 1 min; 15 deg

h. 23 m/min; 1 min; 15 deg

i. 24 m/min; 1 min; 15 deg

j. +1 m/min; 1 min; 15 deg until exhaustion (Exhaustion refers to the point at which mice are incapable of continued running for 30 seconds despite persistent coaxing)

The exercise was done in alphabetical order as shown where mice were started with (a) till (j). The 10 different combinations are used one after the other in a series which increases the difficulty for the mice so that we can take note of exhaustion speed and time to exhaustion.

At study termination, blood sera were obtained through cardiac puncture and natural clotting at room temperature for 30 min. Sera were isolated by centrifuging at 2000 x g for 10 min at 4 °C and kept at -80 °C until further analysis.

Myotube Differentiation and Preconditioning Paradigm

The collection of PEMF-conditioned media (pCM) from C2C12 suspension cells was previously described [3]. Two separate sets of fully differentiated myotubes were prepared beforehand, to be used on days for recipient differentiating myoblast sister cultures. Myotubes, adhered and suspension myoblasts in fresh DMEM were exposed to 1.5 mT PEMFs for 10 min in the downward (pCM, down) direction to produce preconditioning media. cCM (constitutive release, non-PEMF) was produced from unexposed myotubes, adhered and suspension myoblasts. After 1 h of post-magnetic stimulation, isolated cCM or pCM from these myotubes and myoblasts were supplemented with 2% horse serum (Hyclone; Thermo Fisher Scientific) to generate differentiation media for early differentiating myoblast sister cultures. On the final day of differentiation (day 8), these myotubes were either exposed to PEMF (downward) or not, and the resultant cCM or pCM were collected 6 h later for analysis on breast cancer cells.

Effect of gentamicin antibiotics on the efficacy of pCM

Secretome obtained from myotubes that underwent pCM preconditioning and final PEMF exposure was added with 1X Gentamicin (1 mg/ml; working concentration) (Sigma-Aldrich, G1264). The cCM condition was used as a reference scenario. The secretome obtained was then used as cell culture media for cell count and migration assay on MCF-7 and 67NR breast cancer cells to ascertain the anticancer effects of preconditioning pCM and antibiotic effect.

References

1. Tai, Y.K., C. Ng, K. Purnamawati, J.L.Y. Yap, J.N. Yin, C. Wong, et al., Magnetic fields modulate metabolism and gut microbiome in correlation with Pgc-1alpha expression: Follow-up to an in vitro magnetic mitohormetic study. FASEB J, 2020. 34(8): p. 11143-11167. doi: 10.1096/fj.201903005RR.

2. Petrosino, J. M., Heiss, V. J., Maurya, S. K., Kalyanasundaram, A., Periasamy, M., LaFountain, R. A., Wilson, J. M., Simonetti, O. P., & Ziouzenkova, O. (2016). Graded Maximal Exercise Testing to Assess Mouse Cardio-Metabolic Phenotypes. PloS one, 11(2), e0148010. doi: 10.1371/journal.pone.0148010

3. Wong CJK, Tai YK, Yap JLY, Fong CHH, Loo LSW, Kukumberg M, et al. Brief exposure to directionally-specific pulsed electromagnetic fields stimulates extracellular vesicle release and is antagonized by streptomycin: A potential regenerative medicine and food industry paradigm. Biomaterials. 2022;287:121658. doi: 10.1016/j.biomaterials.2022.121658.
